# Intrapulmonary Bronchogenic Cyst Adjacent to the Inferior Pulmonary Ligament Mimicking a Paraesophageal Mediastinal Cyst: A Case Report

**DOI:** 10.7759/cureus.111844

**Published:** 2026-06-30

**Authors:** Toshiyuki Shima

**Affiliations:** 1 Thoracic Surgery, Ashikaga Red Cross Hospital, Tochigi, JPN

**Keywords:** bronchogenic cyst, diagnostic pitfall, inferior pulmonary ligament, intrapulmonary bronchogenic cyst, mediastinal cyst, paraesophageal lesion, thoracoscopic surgery, uniportal vats

## Abstract

Bronchogenic cysts are rare congenital foregut malformations that most commonly arise in the mediastinum. Intrapulmonary bronchogenic cysts are less frequent and may present diagnostic challenges when located adjacent to mediastinal structures. We report a case of an intrapulmonary bronchogenic cyst that closely mimicked a paraesophageal mediastinal cyst on preoperative imaging. A 72-year-old man was referred to our department after a cystic lesion was incidentally detected during a routine health examination. CT demonstrated a 37-mm well-circumscribed cystic lesion in the right lower thorax. The lesion was located between the esophagus, inferior vena cava, vertebral body, and diaphragm. MRI revealed a well-circumscribed cystic lesion with features suggestive of benignity. Based on these findings, a mediastinal bronchogenic cyst or esophageal duplication cyst was suspected. Uniportal video-assisted thoracoscopic surgery was planned for resection of the presumed mediastinal cyst. Intraoperatively, the lesion was found to originate from the right lower lobe adjacent to the inferior pulmonary ligament rather than from the mediastinum. Wedge resection of the affected lung was performed through a single 4-cm incision. Histopathological examination demonstrated ciliated respiratory epithelium, bronchial glands, and cartilage, confirming the diagnosis of an intrapulmonary bronchogenic cyst. The postoperative course was uneventful. This case highlights the difficulty of accurately determining the anatomical origin of cystic lesions arising adjacent to the inferior pulmonary ligament. Intrapulmonary bronchogenic cysts should be considered in the differential diagnosis of paraesophageal mediastinal cysts, particularly when lesions are located near the inferior pulmonary ligament, even when CT and MRI findings strongly suggest a mediastinal origin.

## Introduction

Bronchogenic cysts are rare congenital foregut malformations resulting from abnormal budding of the primitive tracheobronchial tree during embryonic development [1]. They occur predominantly in the mediastinum, whereas approximately 20-30% arise within the pulmonary parenchyma [2-4]. Although many bronchogenic cysts are asymptomatic and incidentally detected on imaging studies, surgical resection is generally recommended because imaging alone cannot always establish a definitive diagnosis and because complications such as infection, hemorrhage, compression of adjacent structures, and, rarely, malignant transformation have been reported [2-6]. Bronchoscopy and endobronchial ultrasound-guided fine-needle aspiration have also been reported as alternative diagnostic modalities in selected patients, particularly when tissue confirmation is required, or airway compression is suspected [7]. However, these techniques may not reliably determine the anatomical origin of cystic lesions adjacent to the inferior pulmonary ligament.

CT and MRI play important roles in the evaluation of cystic thoracic lesions. MRI is particularly useful for characterizing cystic contents and differentiating benign from solid lesions; however, accurately determining the anatomical origin of cysts located adjacent to the inferior pulmonary ligament and paraesophageal region remains challenging [8-10]. Such lesions may closely mimic mediastinal cysts, including bronchogenic cysts and esophageal duplication cysts [9,10]. We report a rare case of an intrapulmonary bronchogenic cyst arising adjacent to the inferior pulmonary ligament that was preoperatively diagnosed as a paraesophageal mediastinal cyst and successfully resected using uniportal video-assisted thoracoscopic surgery (VATS).

## Case presentation

A 72-year-old man was referred to our department after a cystic lesion was detected on a routine chest radiograph during a health screening examination. He was asymptomatic. On admission, his blood pressure was 157/94 mmHg, heart rate was 93 beats/min, respiratory rate was 18 breaths/min, body temperature was 35.9°C, and oxygen saturation was 99% on room air. Physical examination was otherwise unremarkable. Initial laboratory investigations, including complete blood count, serum biochemistry, inflammatory markers, carcinoembryonic antigen (CEA), and soluble interleukin-2 receptor (sIL-2R), were all within normal limits (Table 1). His medical history was notable for benign prostatic hyperplasia, hypertension, dyslipidemia, multiple hepatic cysts, and bilateral renal cysts. He had no history of diabetes mellitus.

**Table 1:** Preoperative laboratory findings AST: aspartate aminotransferase, ALT: alanine aminotransferase, CEA: carcinoembryonic antigen, sIL-2R: soluble interleukin-2 receptor

Parameter	Value	Normal range
White blood cell count	6.0 × 10³/μL	3.3-8.6 × 10³/μL
Hemoglobin	15.1 g/dL	13.7-16.8 g/dL
Platelet count	168 × 10³/μL	158-348 × 10³/μL
AST	20 U/L	13-30 U/L
ALT	19 U/L	10-42 U/L
Creatinine	0.76 mg/dL	0.65-1.07 mg/dL
C-reactive protein	<0.10 mg/dL	0.00-0.14 mg/dL
CEA	1.8 ng/mL	0.00-5.01 ng/mL
sIL-2R	438 U/mL	157-474 U/mL

Contrast-enhanced CT demonstrated a 37-mm well-circumscribed cystic lesion in the right lower thorax. The lesion was located adjacent to the lower thoracic esophagus and appeared to be surrounded by the esophagus, inferior vena cava, vertebral body, and diaphragm (Figure 1). Coronal and sagittal reconstructed images demonstrated that the lesion was situated in the paraesophageal region near the inferior pulmonary ligament, making it difficult to determine whether the lesion originated from the mediastinum or the lung parenchyma.

**Figure 1:**
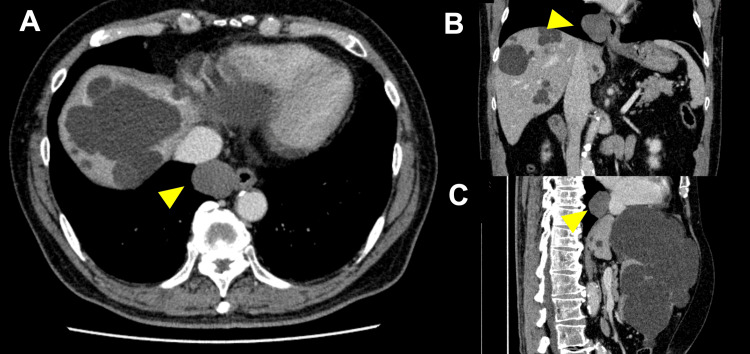
Contrast-enhanced CT findings (A) Axial CT image showing a 37-mm well-circumscribed cystic lesion (arrowhead) adjacent to the lower thoracic esophagus. (B) Coronal CT image demonstrating the lesion (arrowhead) in the paraesophageal region near the inferior pulmonary ligament. (C) Sagittal CT image showing the lesion (arrowhead) located between the esophagus, inferior vena cava, vertebral body, and diaphragm, making its anatomical origin difficult to determine preoperatively. CT: computed tomography

MRI revealed a well-defined cystic lesion showing isointense signal relative to skeletal muscle on T1-weighted imaging and high signal intensity on T2-weighted imaging (Figure 2). The lesion exhibited minimal contrast enhancement, and no evidence of invasion into adjacent structures was identified. Based on the anatomical location and imaging characteristics, a benign mediastinal cyst was suspected. The differential diagnosis included a bronchogenic cyst and an esophageal duplication cyst.

**Figure 2:**
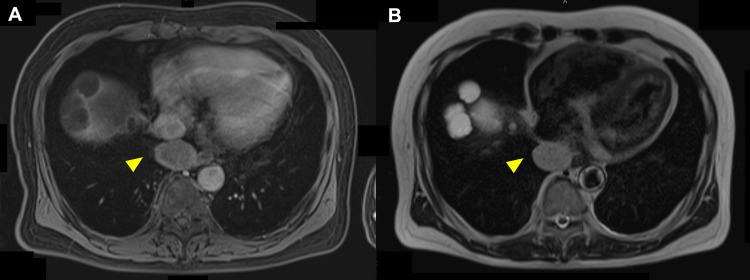
MRI findings (A) Axial contrast-enhanced T1-weighted image showing a well-defined cystic lesion (arrowhead) with signal intensity approximately isointense to skeletal muscle. (B) Axial T2-weighted image demonstrating markedly high signal intensity of the lesion (arrowhead), consistent with a benign cystic lesion. MRI: magnetic resonance imaging

Because a definitive diagnosis could not be established radiologically, surgical resection was planned. Uniportal VATS was performed through a 4-cm incision in the eighth intercostal space. Intraoperatively, the lesion was initially approached as a mediastinal cyst. However, following right lower lobe cranial mobilization, the lesion moved synchronously with the surrounding lung parenchyma. It appeared continuous with adjacent pulmonary tissue, indicating an intrapulmonary origin rather than a mediastinal origin (Figure 3). Based on this finding, wedge resection of the involved lung was performed, and the lesion was completely excised. The operative time was 156 minutes, with minimal blood loss.

**Figure 3:**
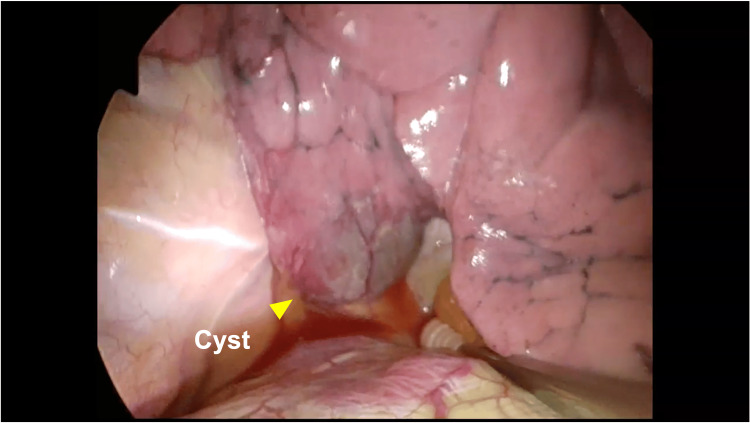
Intraoperative thoracoscopic findings demonstrating the pulmonary origin of the lesion Thoracoscopic examination revealed a cystic lesion adjacent to the inferior pulmonary ligament. Following cranial mobilization of the right lower lobe, the lesion moved synchronously with the surrounding lung parenchyma and appeared continuous with adjacent pulmonary tissue, indicating an intrapulmonary rather than mediastinal origin.

Gross examination demonstrated a collapsed unilocular cystic lesion measuring approximately 37 mm in diameter within the resected wedge specimen (Figure 4). Histopathological examination revealed a cyst lined by ciliated respiratory epithelium and containing bronchial glands and cartilage within the cyst wall (Figure 5). These findings confirmed the diagnosis of an intrapulmonary bronchogenic cyst.

**Figure 4:**
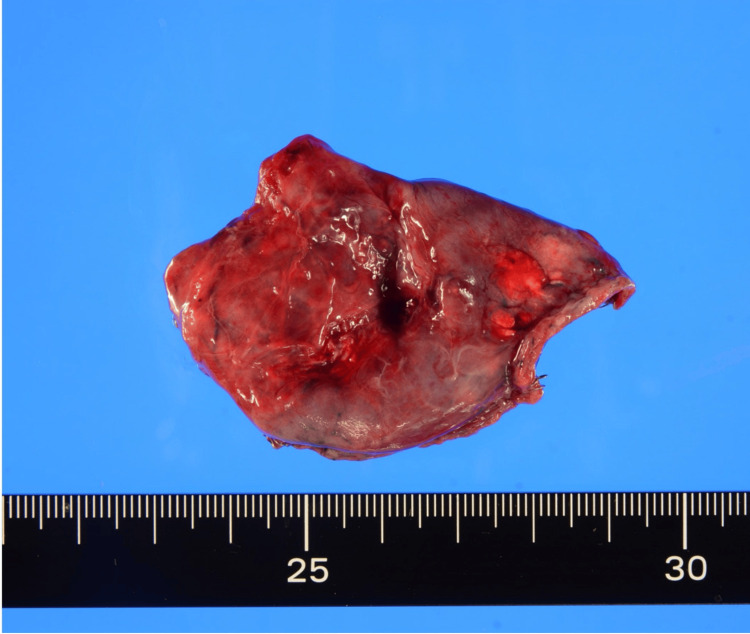
Gross pathological specimen Macroscopic examination of the resected wedge specimen demonstrated a collapsed intrapulmonary cystic lesion measuring approximately 37 mm in diameter. The cyst was collapsed because its contents had been evacuated following inadvertent rupture during surgical resection.

**Figure 5:**
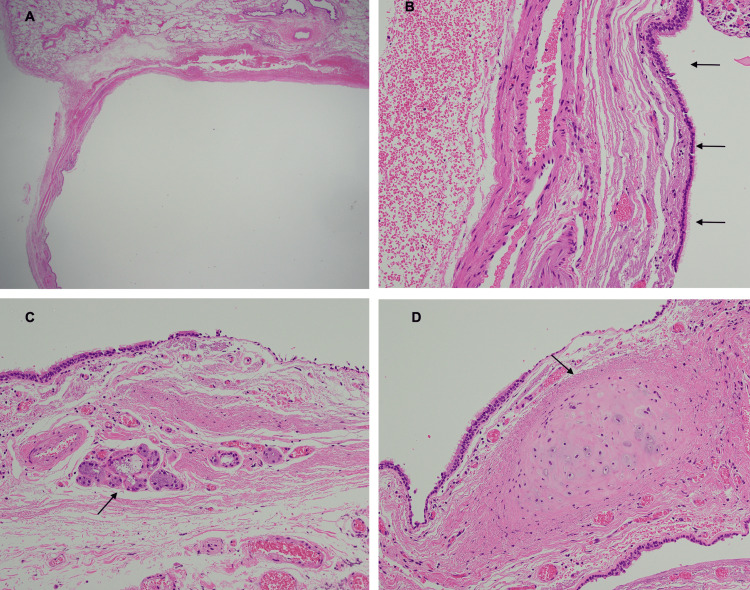
Histopathological findings of the resected cyst (hematoxylin and eosin staining) (A) Low-power view (×12.5) demonstrating the cyst wall. (B) High-power view (×200) showing ciliated respiratory epithelium lining the cyst (black arrows). (C) High-power view (×200) demonstrating bronchial glands within the cyst wall (black arrow). (D) High-power view (×200) showing cartilage within the cyst wall (black arrow), confirming the diagnosis of an intrapulmonary bronchogenic cyst.

The postoperative course was uneventful, and the patient was discharged without complications. At the one-month follow-up, the patient remained asymptomatic. Follow-up CT demonstrated complete resection of the lesion, with no evidence of residual or recurrent cysts (Figure 6).

**Figure 6:**
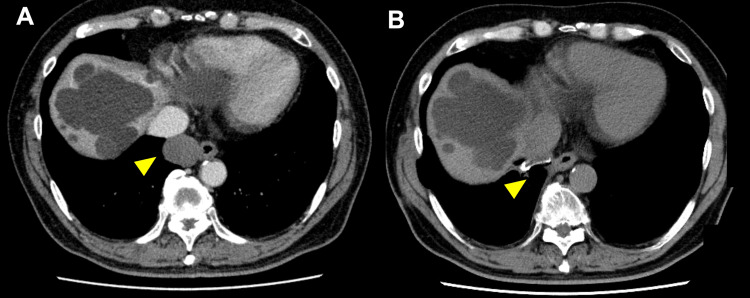
Comparison of preoperative and postoperative CT findings (A) Preoperative axial contrast-enhanced CT demonstrating a 37-mm cystic lesion adjacent to the lower thoracic esophagus (arrowhead). (B) Axial CT obtained one month after surgery showing complete resection of the lesion. The arrowhead indicates the postoperative staple line. No residual or recurrent cystic lesion was identified. CT: computed tomography

## Discussion

Bronchogenic cysts are congenital foregut malformations that arise from abnormal budding of the primitive tracheobronchial tree during embryonic development [1]. Approximately 70-80% occur in the mediastinum, whereas only 20-30% arise within the pulmonary parenchyma [2-4]. Although these lesions are usually benign and remain asymptomatic, they may occasionally cause serious or even life-threatening complications [11]. Surgical resection is generally recommended because imaging alone cannot always establish a definitive diagnosis and because infection, hemorrhage, compression of adjacent structures, and, rarely, malignant transformation have been reported [2-6].

The present case highlights the diagnostic challenge of determining the anatomical origin of cystic lesions arising adjacent to the inferior pulmonary ligament. On contrast-enhanced CT, the lesion appeared to be located between the esophagus, inferior vena cava, vertebral body, and diaphragm, suggesting a paraesophageal mediastinal cyst. MRI findings were also consistent with a benign cystic lesion, demonstrating low-to-intermediate signal intensity on T1-weighted imaging and marked hyperintensity on T2-weighted imaging, without evidence of invasion. Despite excellent tissue characterization, MRI could not accurately determine whether the lesion originated in the mediastinum or the lung parenchyma. This case demonstrates that even comprehensive preoperative imaging may be insufficient to determine the anatomical origin of cystic lesions located near the inferior pulmonary ligament [9,10].

Only a limited number of reports have described intrapulmonary bronchogenic cysts mimicking mediastinal cystic lesions. Takeichi et al. reported an intrapulmonary bronchogenic cyst that was preoperatively diagnosed as a middle mediastinal cystic tumor and successfully treated by thoracoscopic segmentectomy [12]. Our case differs in that the lesion arose immediately adjacent to the inferior pulmonary ligament and closely mimicked a paraesophageal mediastinal cyst. To our knowledge, the diagnostic value of intraoperative assessment of lesion mobility for distinguishing intrapulmonary from mediastinal cysts has rarely been emphasized.

An important clinical message from the present case is the value of careful intraoperative assessment of lesion mobility. The lesion was initially approached as a mediastinal cyst based on preoperative imaging findings. However, following cranial mobilization of the lower lobe, the lesion moved synchronously with the surrounding lung parenchyma and appeared continuous with adjacent pulmonary tissue. This simple intraoperative finding immediately suggested an intrapulmonary origin and prompted conversion from mediastinal cyst excision to pulmonary wedge resection. Careful assessment of lesion mobility may therefore provide an important diagnostic clue when the origin of paraesophageal cystic lesions remains uncertain.

Uniportal VATS was particularly advantageous in this case. The magnified thoracoscopic view enabled meticulous dissection around the inferior pulmonary ligament and paraesophageal region while preserving surrounding structures. Furthermore, the minimally invasive approach enabled careful evaluation of the anatomical relationship between the lesion and the adjacent lung parenchyma prior to resection. Thus, uniportal VATS served not only as a therapeutic approach but also as an effective diagnostic modality for clarifying the lesion’s true anatomical origin [13,14].

Histopathological examination demonstrated the characteristic triad of ciliated respiratory epithelium, bronchial glands, and cartilage within the cyst wall, establishing the diagnosis of an intrapulmonary bronchogenic cyst and excluding other congenital foregut cysts such as esophageal duplication cysts [8].

The postoperative course was uneventful. At the one-month follow-up, the patient remained asymptomatic, and follow-up CT demonstrated complete resection without residual or recurrent cystic lesions. Although this report describes a single patient, it highlights an important clinical message. Intrapulmonary bronchogenic cysts adjacent to the inferior pulmonary ligament should be considered in the differential diagnosis of paraesophageal mediastinal cysts, even when CT and MRI strongly suggest a mediastinal origin. Careful intraoperative assessment of lesion mobility may provide a valuable clue to the correct diagnosis.

## Conclusions

We encountered a rare case of an intrapulmonary bronchogenic cyst arising adjacent to the inferior pulmonary ligament that mimicked a paraesophageal mediastinal cyst. Despite detailed preoperative CT and MRI evaluation, the anatomical origin of the lesion could not be accurately determined until surgical exploration. Bronchogenic cysts should be considered in the differential diagnosis of cystic thoracic lesions, particularly when paraesophageal cystic lesions are located near the inferior pulmonary ligament. Careful intraoperative assessment of lesion mobility remains essential for establishing the correct diagnosis, and uniportal VATS provides an effective minimally invasive approach for both diagnosis and treatment.
